# Effects of Blending Temperature and Time on Interfacial Diffusion Behavior and Fatigue Response of Virgin–Aged Asphalt Binders

**DOI:** 10.3390/ma19183977

**Published:** 2026-09-19

**Authors:** Jiangtao Fan, Jing Zhang, Xiaodong Xie, Yu Zhang, Zhenxun Wei

**Affiliations:** 1 School of Civil Aviation, XiHang University, Xi’an 710077, China; 2020021070@chd.edu.cn (J.F.); zyu@chd.edu.cn (Y.Z.); 2 Xinjiang Transportation Science Research Institute Co., Ltd., Urumqi 830099, China; 202407009@xaau.edu.cn; 3 Xinjiang Key Laboratory of Highway Engineering Technology in Arid Desert Areas, Urumqi 830099, China; xiaodong3056@163.com

**Keywords:** RAP, virgin and aged asphalt binders, interfacial diffusion, fatigue performance, molecular dynamics

## Abstract

To elucidate the diffusion behavior between virgin and aged asphalt binders and its relationship with fatigue performance, a concentric-circle blending system with a well-defined initial interface was constructed. By varying the blending temperature and time, the interfacial blending process was characterized from the perspectives of macroscopic performance, component migration, and molecular motion using dynamic shear rheometry, linear amplitude sweep, thin-layer chromatography with flame ionization detection, fluorescence microscopy, and molecular dynamics simulations. The results showed that thermal exposure produced a continuous interfacial transition region between the virgin and aged asphalt binders. Fatigue life generally increased as the sampling position shifted from the aged-binder side toward the virgin-binder side. At 5% strain, increasing the blending temperature from 135 to 165 °C increased the interfacial fatigue life by 173.8%, while extending the blending time from 15 to 60 min increased the fatigue life by 96.6%. The SARA fractions and fluorescence morphology results indicated that thermal exposure promoted component migration and redistribution on both sides of the interface, causing the initially distinct interface to gradually evolve into a continuous transition region. Molecular simulations further demonstrated that increasing temperature significantly enhanced the molecular mobility of all components and expanded the spatial overlap between the virgin and aged asphalt binders in the interfacial region. These results indicate that an increased extent of diffusion corresponds to an increase in fatigue life within the interfacial region and that the blending state between virgin and aged asphalt binders is an important factor affecting the local fatigue performance of recycled asphalt binder.

## 1. Introduction

As road infrastructure gradually shifts from large-scale construction to maintenance and recycling, the high-value recycling of reclaimed asphalt pavement (RAP) has become an important approach to conserving resources and reducing carbon emissions in pavement engineering. RAP contains not only reusable aggregates but also a substantial proportion of aged asphalt binder. Therefore, improving the effective utilization of aged asphalt binder during recycling is essential for further increasing RAP content. However, during actual hot recycling, the virgin asphalt binder and the aged asphalt binder on RAP surfaces do not undergo instantaneous and complete mixing under idealized conditions. Instead, they experience a dynamic process involving thermal softening, interfacial contact, molecular diffusion, component migration, and gradual homogenization, with the final state generally lying between no blending and complete blending [1,2]. Such incomplete blending not only affects the actual composition of the effective binder in the recycled system but also produces local differences in material composition and mechanical properties, thereby affecting the macroscopic performance and durability of recycled asphalt materials. Therefore, clarifying the interfacial blending behavior between virgin and aged asphalt binders and its effects on performance is fundamental to reasonably evaluating the effective utilization of RAP and optimizing hot-recycling processes. Recent reviews have also identified the accurate characterization of the degree of blending and the multiscale clarification of its formation mechanism as core issues in recycled asphalt research.

Blending between virgin and aged asphalt binders is jointly affected by temperature, conditioning time, RAP content, material composition, and mixing method [3]. Previous studies have generally shown that increasing the temperature and extending the thermal conditioning time can promote aged-binder activation and interfacial mass transfer, although different degrees of heterogeneity usually remain in actual systems. Alavi et al. [4] used virgin–aged binder contact specimens and concentric-circle specimens to investigate interfacial diffusion at 135 and 165 °C under different conditioning times and found that both increasing the temperature and extending the conditioning time were beneficial to increasing the degree of blending. Yao et al. [5] analyzed blending behavior under multiple influencing factors from the perspectives of material composition and the mixing process. Zou et al. [6] further found that virgin and aged asphalt binders continued to diffuse during storage and transportation after mixing, demonstrating the pronounced time dependence of blending. Meanwhile, fluorescence microscopy, confocal microscopy, scanning electron microscopy (SEM), and atomic force microscopy (AFM) have increasingly been used to directly characterize interfacial evolution and the degree of blending between virgin and aged asphalt binders [7,8,9,10], allowing blending research to progress from bulk performance evaluation to spatial characterization of the interface. From a microscopic perspective, blending between virgin and aged asphalt binders is intrinsically related to the thermal motion of molecules on both sides of the interface and their migration across the interface. Molecular dynamics simulations provide a molecular-scale approach for investigating interfacial diffusion through parameters such as mean square displacement (MSD), diffusion coefficients, and concentration or density distributions [11]. Ge et al. [12] identified the blending region between virgin and aged asphalt binders based on relative density distributions and showed that increasing temperature promoted interfacial diffusion and improved interfacial interactions. Subsequent studies have also demonstrated that temperature, time, and aging degree significantly affect molecular migration behavior [13,14,15]. Moreover, diffusion between virgin and aged asphalt binders is accompanied by the spatial redistribution of saturates, aromatics, resins, and asphaltenes (SARA), whose composition is closely related to the colloidal structure and viscoelasticity of asphalt binder. Jiang et al. [16] reported that different proportions of the SARA fractions significantly altered the rheological and fatigue responses of asphalt binder, suggesting that component redistribution may represent an important link between interfacial diffusion and changes in macroscopic performance. Nevertheless, existing studies have mainly focused on the degree of blending, interfacial morphology, bulk rheological performance, and molecular diffusion behavior, whereas relatively limited attention has been paid to the coordinated evolution of composition and performance at different spatial locations during blending. In particular, the effect of blending between virgin and aged asphalt binders on fatigue performance remains insufficiently understood. The linear amplitude sweep (LAS) test has been widely used to evaluate the fatigue damage behavior of aged and recycled asphalt binders [17,18,19], and previous studies have shown that the degree of blending may significantly affect the fatigue performance of systems containing high RAP contents [20]. However, most studies have been based on fully blended recycled asphalt binders or the bulk performance of asphalt mixtures, making it difficult to reveal the spatial variation in fatigue performance from the aged-binder side, through the interfacial region, to the virgin-binder side. Therefore, establishing a direct relationship between blending behavior and fatigue performance from the perspective of interfacial evolution is important for gaining an in-depth understanding of the service performance of recycled asphalt binders.

Based on these considerations, this study constructed a concentric-circle blending system of virgin and aged asphalt binders with a well-defined initial interface. By controlling the blending temperature and time, the diffusion and blending behaviors of the virgin and aged asphalt binders were investigated at different scales in terms of interfacial morphology, component composition, rheological performance, and molecular motion. Different radial sampling positions were established along the normal direction of the initial interface. DSR has long been widely used to characterize the viscoelastic and rheological properties of asphalt binders under different temperatures, frequencies, and testing conditions [21,22]. Fluorescence microscopy and thin-layer chromatography with flame ionization detection (TLC-FID) were used to characterize interfacial evolution and spatial variations in the SARA fractions, while dynamic shear rheometry (DSR) was used to analyze the rheological characteristics at different blending positions. On this basis, the LAS test was employed to evaluate changes in fatigue life at different radial positions and under different temperature and time conditions, with particular emphasis on the intrinsic relationships among the extent of diffusion, interfacial blending state, and fatigue performance. Molecular dynamics simulations were also conducted to analyze the molecular motion of individual components and their migration across the interface at different temperatures, thereby explaining the effect of temperature on the diffusion behavior between virgin and aged asphalt binders at the molecular scale. This study aims to reveal the diffusion and blending mechanisms between virgin and aged asphalt binders from both experimental and molecular-scale perspectives and to further clarify how changes in interfacial composition and state during diffusion affect the resistance of asphalt binder to fatigue damage, thereby providing a theoretical basis for evaluating the effective degree of blending of aged asphalt binder in RAP and its effects on fatigue performance.

## 2. Materials and Methods

### 2.1. Materials

#### 2.1.1. Base Asphalt Binder

The asphalt binder used in this study was a commercially available Grade 90 base asphalt binder purchased from a local supplier in China, designated as VA. Its physical properties were tested in accordance with the Standard Test Methods of Asphalt and Asphalt Mixture for Highway Engineering (JTG 3410-2025) [23]. The test results, which satisfied the specification requirements, are presented in Table 1.

#### 2.1.2. Aged Asphalt Binder

To simulate the long-term aged state of reclaimed asphalt binder in RAP, the Grade 90 base asphalt binder was sequentially subjected to RTFO and PAV aging. Specifically, the RTFO aging test was conducted according to T 0610-2011 in JTG 3410-2025 at 163 °C for 85 min to simulate the short-term aging of asphalt binder during mixing, transportation, and paving. Subsequently, the PAV aging test was conducted according to T 0630-2025 in JTG 3410-2025 [23]. The short-term aged asphalt binder was placed in PAV pans and aged at 100 °C and 2.1 MPa for 20 h to simulate the oxidative hardening of asphalt binder during long-term service. The resulting long-term aged asphalt binder was designated as AA and used to represent the aged asphalt binder phase in RAP.

#### 2.1.3. Concentric-Circle Diffusion Specimens

To characterize the radial diffusion behavior of the virgin and aged asphalt binders under thermal exposure, interfacial diffusion specimens were prepared using a concentric-circle configuration consisting of an aged asphalt binder inner core and a virgin asphalt binder outer ring. The concentric-circle specimens were formed in high-temperature-resistant silicone molds. The outer mold had an inner diameter of 160 mm, the aged asphalt binder inner core had a diameter of 100 mm, and the specimen thickness was 8 mm. Thus, the inner core had a radius of 50 mm, while the virgin asphalt binder outer ring had a radial width of 30 mm. This geometry allowed multiple nonoverlapping sampling regions to be arranged on both sides of the initial interface and ensured that sufficient material was available at each location for subsequent DSR and LAS specimen preparation.

During specimen preparation, the aged asphalt binder was first heated until adequate fluidity was achieved, slowly stirred until homogeneous, and then poured into the inner-core mold with a diameter of 100 mm. The surface was leveled using a preheated scraper. After cooling to room temperature, the specimen was maintained at -5 °C for 30 min to ensure that the aged asphalt binder inner core was fully set. The inner core was subsequently demolded and placed at the geometric center of the outer mold with a diameter of 160 mm using a positioning device. The preheated virgin asphalt binder was slowly poured around the outer perimeter of the inner core to ensure full contact between the two binders along the sidewall while preventing the virgin asphalt binder from covering the upper surface of the inner core. Immediately after forming, the specimen was cooled at −5 °C for 30 min to limit uncontrolled diffusion before the prescribed isothermal treatment.

**Figure 1:**
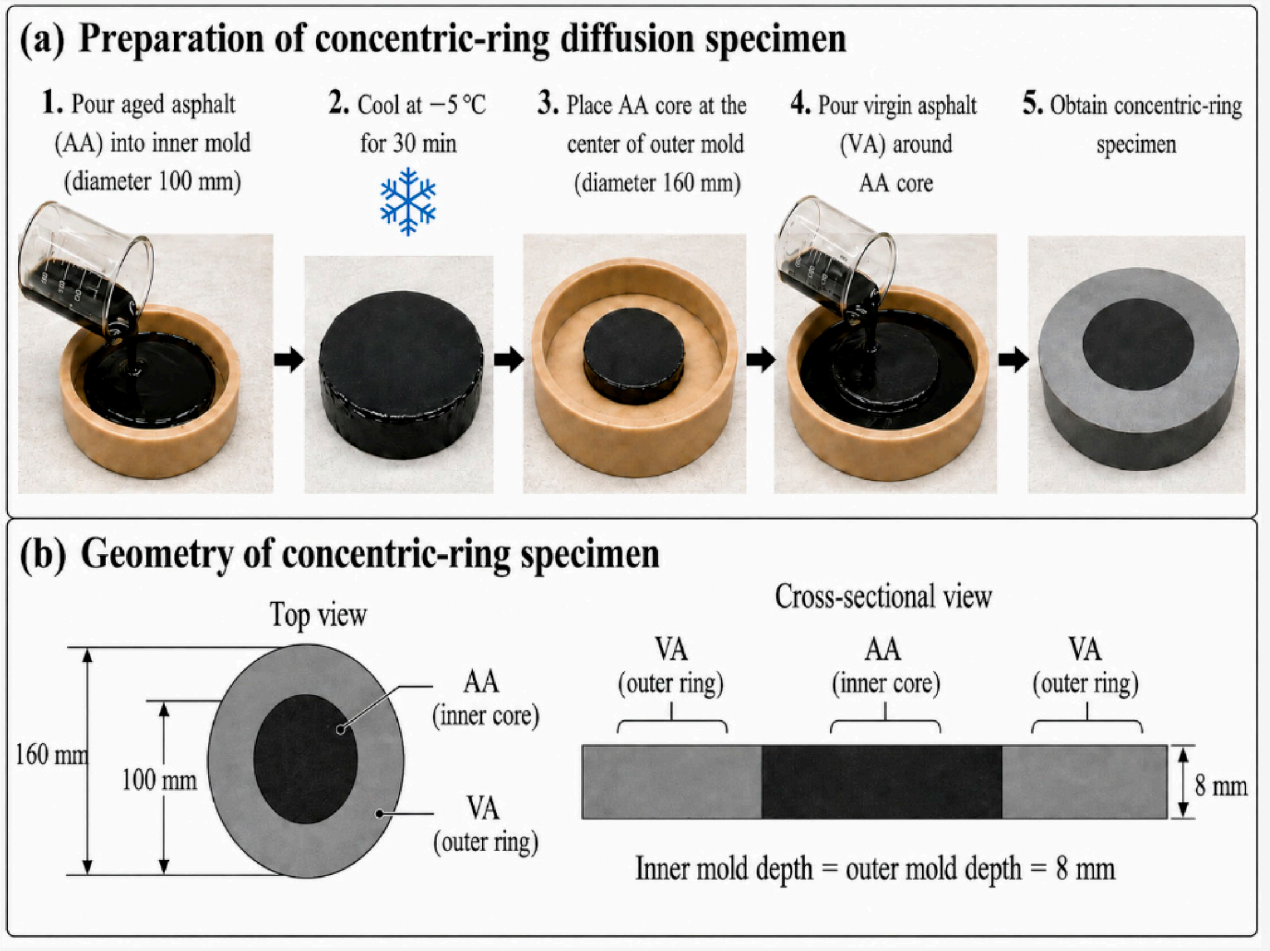
Blending model of virgin and aged asphalt binders.

Two sets of single-factor tests were conducted. For the temperature group, the blending time was fixed at 60 min, and the blending temperatures were 135, 150, and 165 °C. The corresponding specimens were designated D135-60, D150-60, and D165-60, respectively. For the time group, the blending temperature was fixed at 165 °C, and the blending times were 15, 30, and 60 min. The corresponding specimens were designated D165-15, D165-30, and D165-60, respectively. D165-60 was the common condition shared by the temperature- and time-effect tests. The specimen preparation process is illustrated in Figure 1.

#### 2.1.4. Radial Zoning, Sampling, and Specimen Preparation

**Figure 2:**
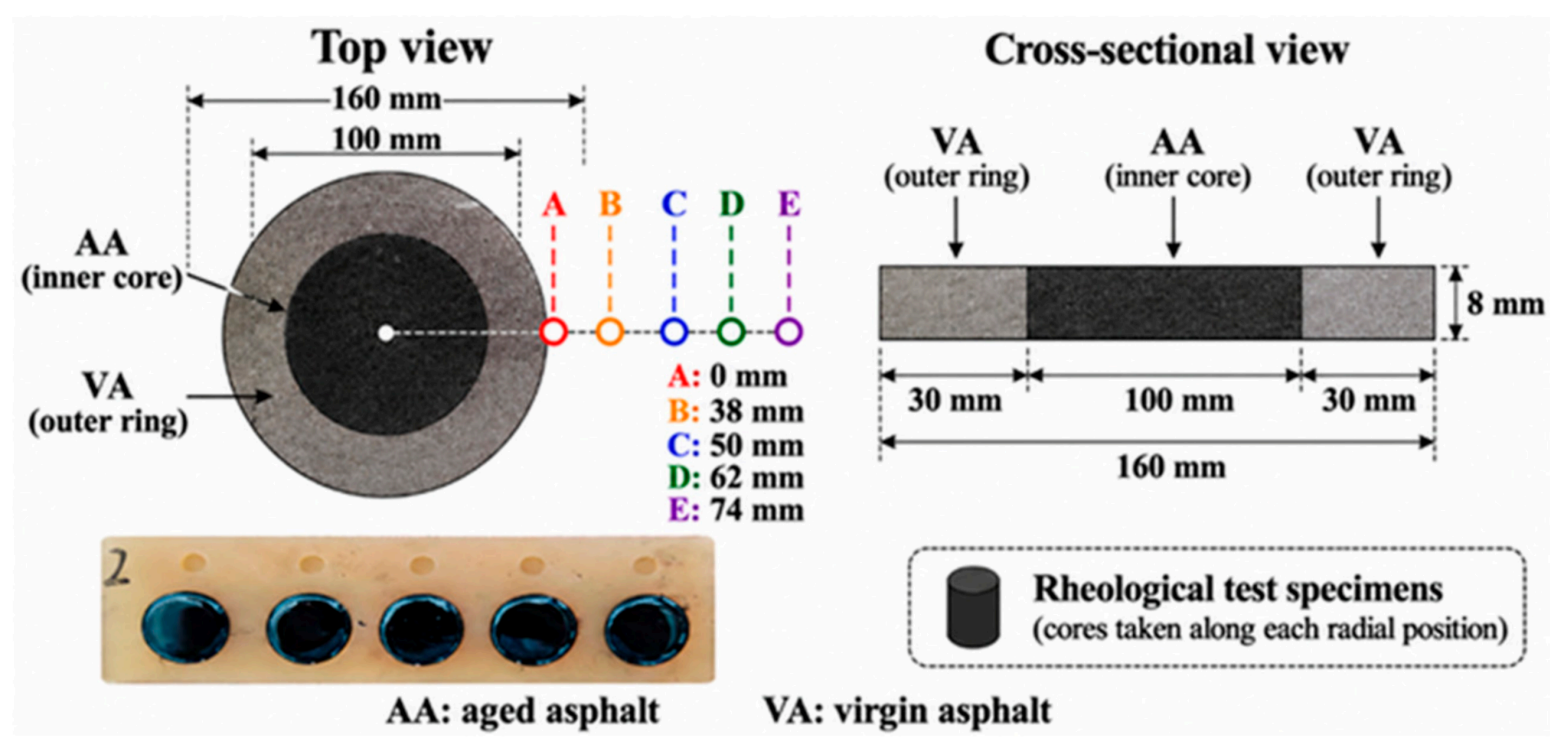
Radial sampling.

After the prescribed isothermal blending treatment, each concentric-circle interfacial diffusion specimen was immediately transferred to an environment at −5 °C and cooled for 30 min to reduce the rate of subsequent molecular migration and preserve the interfacial diffusion state established under the corresponding temperature and time conditions. Five sampling positions, A–E, were then defined along the radial direction under low-temperature conditions, at distances of 0, 38, 50, 62, and 74 mm from the center, respectively. Region A represented the central area of the aged asphalt binder far from the interface; Region B represented the near-interface area on the aged-binder side; Region C was centered at the initial interface radius of 50 mm and covered the range of 45–55 mm; Region D represented the near-interface area on the virgin-binder side; and Region E represented the outer-edge area of the virgin asphalt binder far from the interface. Region A was sampled directly from the specimen center. For Regions B–E, a circular corer with an inner diameter of 10 mm was used to extract three cores at equal angular intervals along the circumference at the corresponding radius. Cores obtained from the same parent specimen and radial position were combined to reduce the influence of local heterogeneity on the test results. Samples from different parent specimens were not mixed, thereby preserving independent experimental replicates. Samples from each position were separately labeled and sealed for storage. Before testing, the samples were briefly softened and formed at the minimum temperature necessary, and all samples underwent the same thermal treatment to minimize the effects of secondary heating on their composition and rheological properties. The five radial positions A–E of specimen D165-60 were used to systematically evaluate the spatial gradient in rheological properties produced by interfacial diffusion, whereas the interface-adjacent regions under the other temperature–time conditions were used to analyze the effects of blending temperature and blending time on the extent of diffusion between the virgin and aged asphalt binders. The sampling process is illustrated in Figure 2.

### 2.2. Experimental Methods

#### 2.2.1. DSR Rheological Test

The DSR temperature-sweep test was conducted with reference to T 0628-2011 in JTG 3410-2025 to evaluate the rheological properties of asphalt binder samples under different blending conditions and at different radial positions. A dynamic shear rheometer (SmartPave 303, Anton Paar GmbH, Graz, Austria) was used to conduct temperature-sweep tests on asphalt binder samples obtained under different blending conditions and from different radial positions. Before the temperature sweep, a strain-sweep test was performed at 46 °C to confirm that the selected strain was within the linear viscoelastic region of all samples. The tests were conducted using 25-mm-diameter parallel plates with a 1-mm gap, a strain of 12%, and a loading frequency of 1.59 Hz. The test temperatures were 46, 52, 58, 64, and 70 °C, and testing at each temperature was initiated after the sample reached thermal equilibrium. The rutting factor was used as the evaluation index to analyze the effects of blending temperature, blending time, and radial position on the high-temperature resistance of the virgin and aged asphalt binders to permanent deformation. Three replicate tests were performed for each sample group, and the results were averaged.

#### 2.2.2. LAS Test

The LAS test was conducted with reference to T 0648-2025 in JTG 3410-2025, and a DSR was used to evaluate the fatigue performance of asphalt binder samples at different radial positions and under different blending conditions. LAS tests were conducted using the DSR on samples obtained from the five radial positions A–E of D165-60 and on samples D135-60-C, D150-60-C, D165-60-C, D165-15-C, and D165-30-C, with VA and AA used as controls. D165-60-C was the common reference sample for the temperature and time groups. The test temperature was 25 °C, and 8-mm-diameter parallel plates with a 2-mm gap were used. After reaching the target temperature of 25 °C, each specimen was held isothermally for at least 10 min. A frequency sweep from 0.2 to 30 Hz was first conducted at a shear strain of 0.1%, followed by a strain sweep at 10 Hz. The strain levels were 0.1%, 1%, and 2–30%, yielding a total of 31 levels, with loading applied for 10 s at each level. The fatigue parameters and fatigue life were calculated from the frequency-sweep and strain-sweep results. The equations used to calculate fatigue life are given below.

The cumulative damage D(t) during the strain sweep was calculated based on viscoelastic continuum damage (VECD) theory:

(1) $$D(t)≅\sum_{i=1}^{N}{\left[\pi G_{1\%}\Upsilon_{i}^{2}(G_{i-1}^{*}sin\delta_{i-1}-G_{i}^{*}sin\delta_{i}\right]}^{\frac{\alpha }{1+\alpha }}{\left(t_{i}-t_{i-1}\right)}^{\frac{1}{1+\alpha }}$$

where $G_{1\%}$ is the initial dynamic shear modulus at the 1% strain stage, $\Upsilon_{i}$ is the shear strain at the corresponding data point, and $t_{i}$ is the loading time. where α=1/m, and m is the slope of the linear relationship between $\log G^{\prime }$ and logω obtained from the frequency-sweep results. A power function was then used to describe the relationship between $G^{*}sin\delta$ and cumulative damage:

(2) $$G^{*}sin\delta =C_{0}-C_{1}D^{C_{2}}$$

where $C_{0}$ is the average value of $\left|G^{*}\right|sin\delta$ within the 0.1% strain interval, and $C_{1}$ and $C_{2}$ are fitting parameters. A 35% reduction in $G^{*}sin\delta$ was adopted as the fatigue damage criterion. The fatigue-model parameter $A_{35}$ and fatigue life $N_{f}$ were calculated using the following equations:

(3) $$D_{f}={\left(\frac{{0.35C}_{0}}{C_{1}}\right)}^{1/C_{2}}$$

(4) $$A_{35}=\frac{f{\left(D_{f}\right)}^{k}}{k{\left(\pi G_{1\%}C_{1}C_{2}\right)}^{\alpha }}\;k=1\;+\;(1-C_{2})\alpha$$

(5) $$N_{f}=A_{35}{\left(\Upsilon_{max}\right)}^{-B}\;B=2\alpha$$

where f is the loading frequency of 10 Hz, $\Upsilon_{max}$ is the prescribed maximum shear strain, and $N_{f}$ is the fatigue life at the corresponding strain level. The $N_{f}$ values at radial positions A–E were compared to analyze the effect of interfacial diffusion between the virgin and aged asphalt binders on spatial differences in fatigue damage performance. To facilitate quantitative comparison among different samples, a maximum shear strain of 5% was selected as a representative strain level, and the corresponding fatigue life, $N_{f,5\%}$, was calculated using the VECD-based fatigue model. It should be noted that the 5% strain was not applied as an independent fixed loading condition during the LAS test, but was used as a prescribed strain level for fatigue-life calculation and comparison.

#### 2.2.3. Thin-Layer Chromatography with Flame Ionization Detection

The asphalt four-component test was conducted with reference to T 0634-2025 (TLC/FID) in JTG 3410-2025 to determine the SARA fraction contents at different radial positions of the D165-60 specimen. The asphalt binder was first dissolved in dichloromethane to prepare a solution with a mass fraction of 1.5%, and 1 μL of the solution was spotted onto a chromarod using a microsyringe. The chromarod was sequentially developed in n-hexane, toluene, and a dichloromethane–methanol mixture with a volume ratio of 95:5 to separate the saturates, aromatics, resins, and asphaltenes, respectively. After development, the chromarod was scanned using an Iatroscan MK-6s TLC–FID ana-lyzer (Mitsubishi Kagaku Iatron, Inc., Tokyo, Japan). The relative content of each fraction was calculated as the percentage of its peak area relative to the total peak area to analyze the radial migration and redistribution of chemical components during interfacial diffusion between the virgin and aged asphalt binders.

#### 2.2.4. Fluorescence Microscopy

An Olympus BX53 fluorescence microscope (Evident Scientific, Tokyo, Japan) was used to observe the interfacial diffusion process between the virgin and aged asphalt binders under thermal exposure. The two binders were prepared as thin layers of uniform thickness and arranged to form a distinct contact interface on a glass slide, after which they were isothermally treated at 165 °C for 0, 30, 60, and 120 s. The specimens were immediately cooled after the prescribed treatment time to preserve the interfacial morphology at the corresponding time. The interfacial region was identified from the difference in fluorescence response between the virgin and aged asphalt binders, and images were acquired using the same magnification, exposure time, and light-source intensity.

### 2.3. Molecular Simulation

#### 2.3.1. Base Asphalt Binder Model

**Figure 3:**
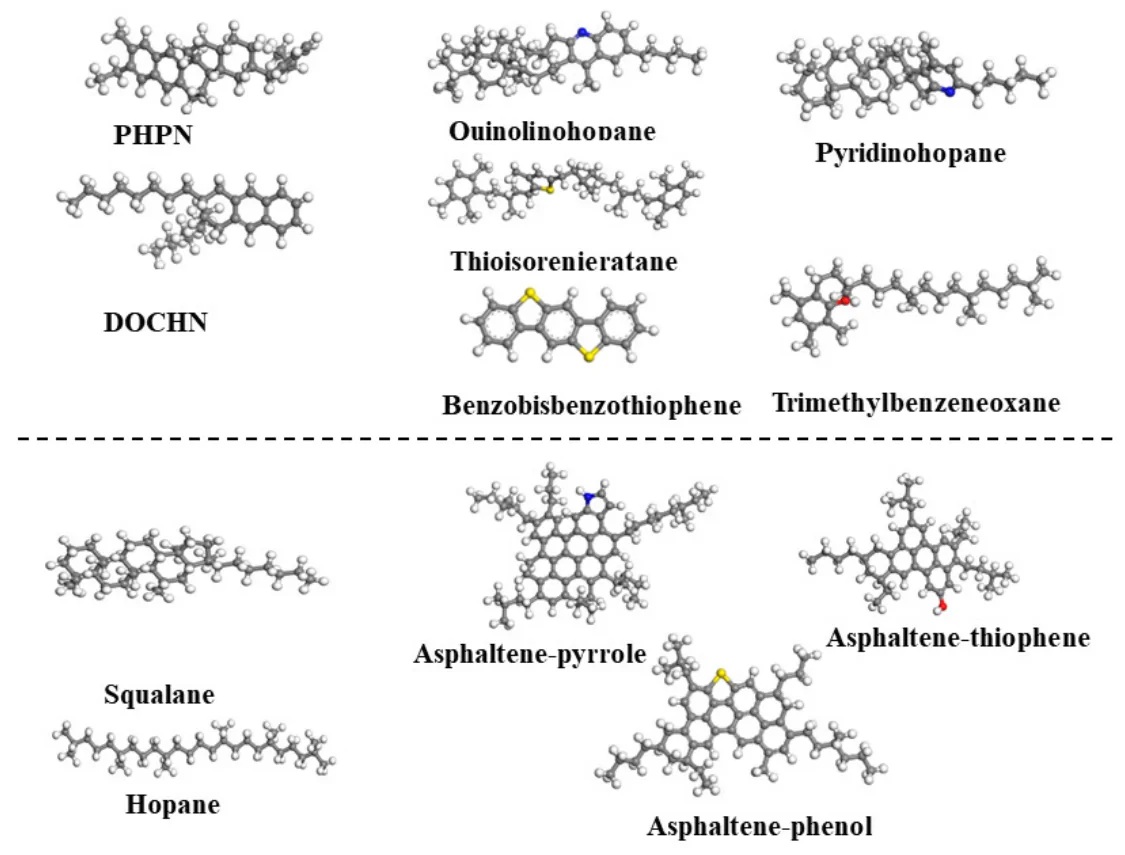
Twelve representative molecular components of the virgin asphalt binder.

The four-component, twelve-molecule asphalt binder model proposed by Li and Greenfield [24] was adopted, and the number of each representative molecule was determined according to the measured four-component contents of the Grade 90 base asphalt binder. A three-dimensional periodic amorphous model was constructed using the Amorphous Cell module in Materials Studio 2020, with the initial density set to 0.1 g/cm^3^ to reduce molecular overlap and local packing. Geometry optimization was then performed using the Forcite module under the COMPASS II force field. The Smart algorithm was employed with a maximum of 50,000 iterations. Electrostatic interactions were calculated using the Ewald method, whereas van der Waals interactions were calculated using the atom-based method with a cutoff radius of 12.5 Å. The cutoff radius represents the spatial cutoff distance used for calculating van der Waals nonbonded interactions. The same cutoff radius was adopted for the aged asphalt binder model and the virgin–aged asphalt binder blending model to ensure consistency of the simulation conditions. After geometry optimization, cyclic annealing between 298.15 and 500 K was performed under the NVT ensemble to improve the molecular configuration and spatial distribution, using a time step of 1.0 fs. After annealing, the model was sequentially equilibrated for 300 ps under the NPT ensemble and for 200 ps under the NVT ensemble at 298.15 K and 1 atm. Temperature was controlled using the Nosé thermostat, and pressure was controlled using the Andersen barostat. Equilibrium was assessed from the variations in system density and energy with time, and the final stable configuration was used as the Grade 90 base asphalt binder model. The structures and numbers of the representative molecules are presented in Figure 3 and Table 2, respectively.

#### 2.3.2. Aged Asphalt Binder Model

**Figure 4:**
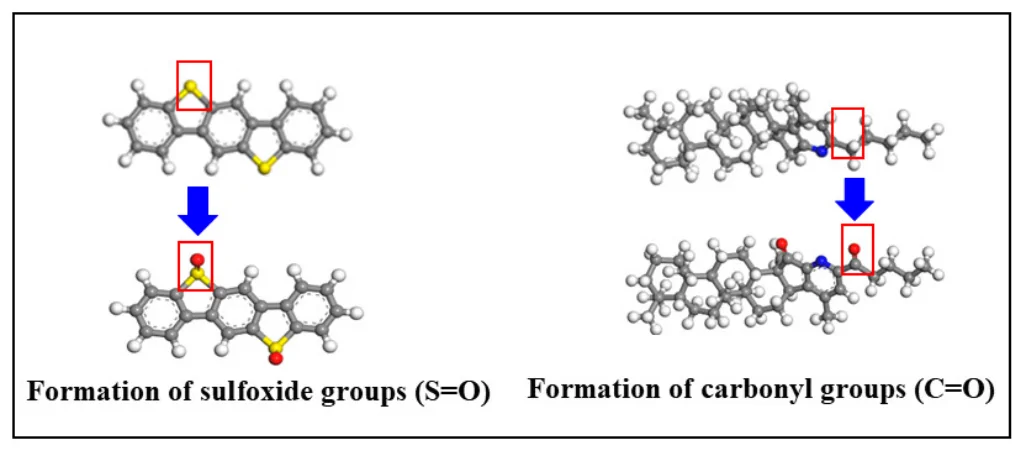
Functional-group changes in the asphalt binder components.

The molecular model of the aged asphalt binder was constructed on the basis of the virgin asphalt binder model. Following the treatment of aged asphalt molecular structures reported by Zhan et al. [25], selected oxidation-prone molecules were modified with func-tional groups, as illustrated in Figure 4. Carbonyl groups were introduced into the side chains of aromatic rings and at benzylic carbon atoms, and selected sulfur-containing structures were oxidized to sulfoxide groups to represent the increases in asphalt binder polarity and oxygen-containing functional groups during thermo-oxidative aging. Meanwhile, the number of each representative molecule was adjusted according to the four-component test results for the aged asphalt binder so that the model composition approximated that of the actual aged asphalt binder.

**Figure 5:**
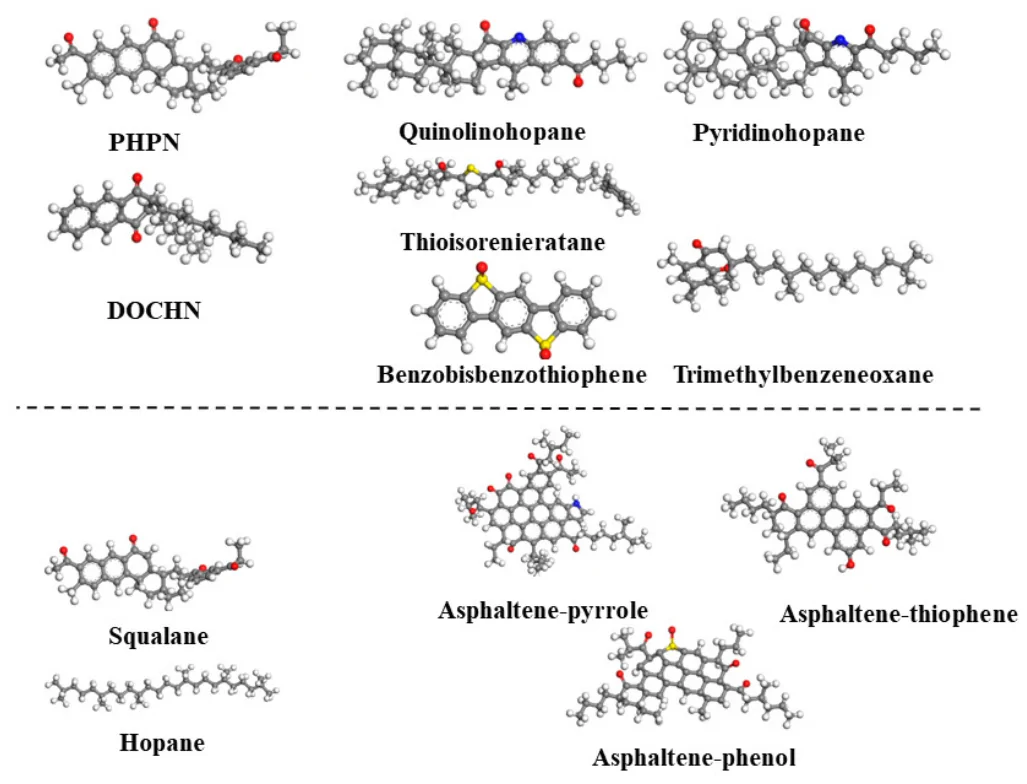
Twelve representative components of the aged asphalt binder.

The four-component model of aged asphalt is shown in Figure 5. The aged asphalt binder model was constructed using the Amorphous Cell module, with the initial density set to 0.1 g/cm^3^ and three-dimensional periodic boundary condi-tions applied. To ensure comparability between the virgin and aged asphalt binder mod-els, the parameters used for geometry optimization, nonbonded-interaction calculations, annealing, and dynamic equilibration were identical to those used for the Grade 90 virgin asphalt binder model. Specifically, the COMPASS II force field, Smart optimization algo-rithm, Ewald method for electrostatic interactions, and atom-based method for van der Waals interactions were used, with a cutoff radius of 12.5 Å. After cyclic annealing, the model was sequentially equilibrated for 300 ps under the NPT ensemble and 200 ps under the NVT ensemble. The final configuration obtained after the density, volume, and energy had stabilized was used as the aged asphalt binder model for the subsequent simulation of interfacial diffusion between the virgin and aged asphalt binders.

#### 2.3.3. Virgin–Aged Asphalt Binder Blending Model

**Figure 6:**
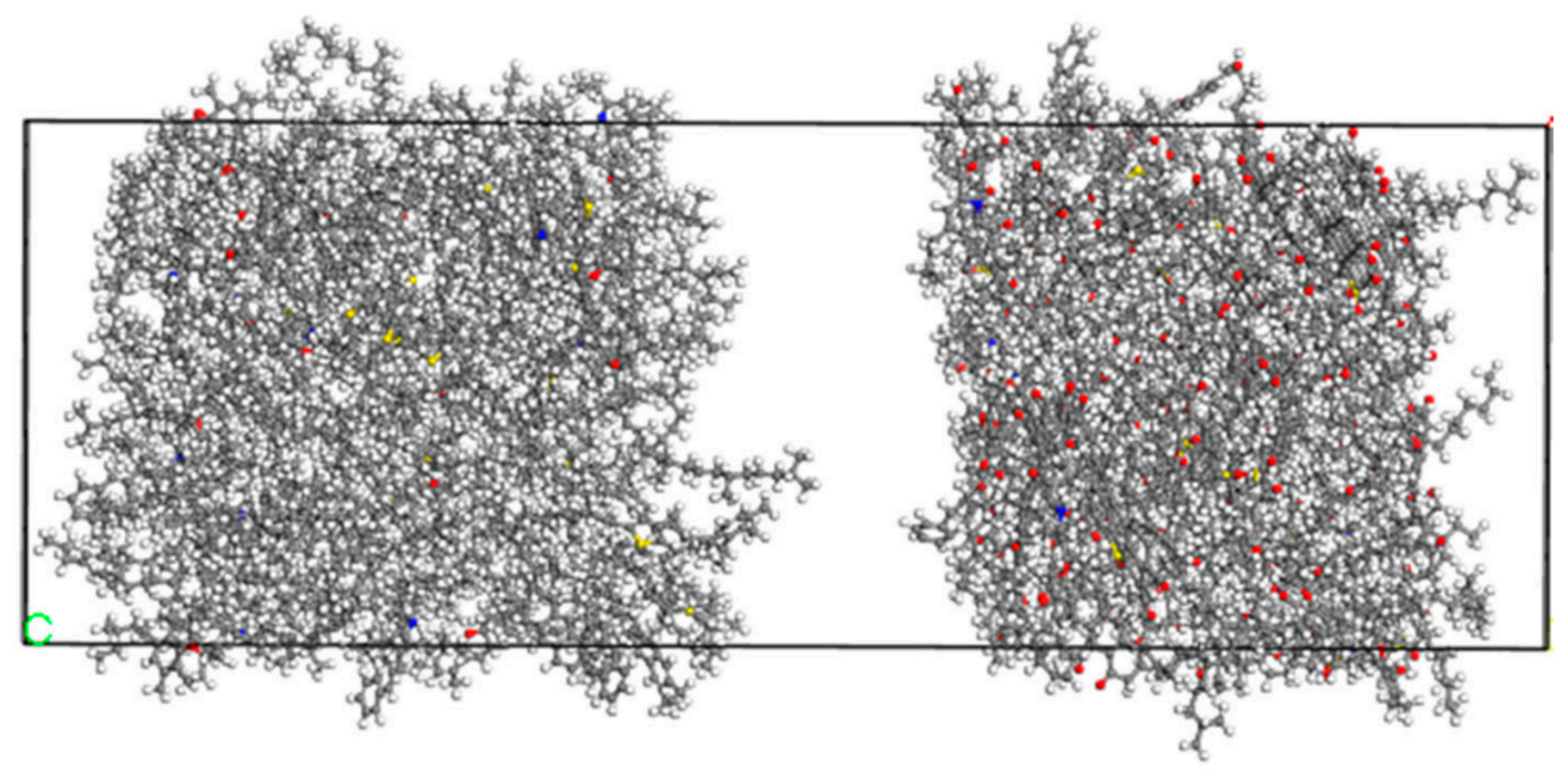
Blending and diffusion model of the virgin and aged asphalt binders.

After the virgin and aged asphalt binder models had been independently equilibrated, the Build Layers tool in Materials Studio 2020 was used to construct a layered model along the Z direction. A virgin asphalt binder–aged asphalt binder bilayer interfacial model was established, with an initial interfacial spacing of 5 Å between the two binder layers to prevent interfacial atomic overlap and provide the space necessary for molecular rear-rangement. This spacing was used only as an initial modeling parameter; no positional constraints were imposed on interfacial molecules during the subsequent optimization and molecular dynamics simulations. The layered interfacial model was used to represent molecular migration driven by the concentration gradient after contact between the virgin and aged asphalt binders. The COMPASS II force field was also used for the blending model. Electrostatic interactions were calculated using the Ewald method, whereas van der Waals interactions were calculated using the atom-based method with a cutoff radius of 12.5 Å. The model was first geometrically optimized using the Smart algorithm to eliminate unreasonable configurations generated during layer assembly. NPT molecular dynamics simulations were subsequently performed for 200 ps at 408.15, 423.15, and 438.15 K, corresponding to 135, 150, and 165 °C, respectively. The system pressure was set to 1 atm, the time step was 1.0 fs, temperature and pressure were controlled using the Nosé thermostat and Andersen barostat, respectively, and the trajectory output interval was 1 ps. The aging asphalt and matrix asphalt diffusion model is shown in Figure 6.

## 3. Results and Discussion

### 3.1. Shear Rheological Test Results

**Figure 7:**
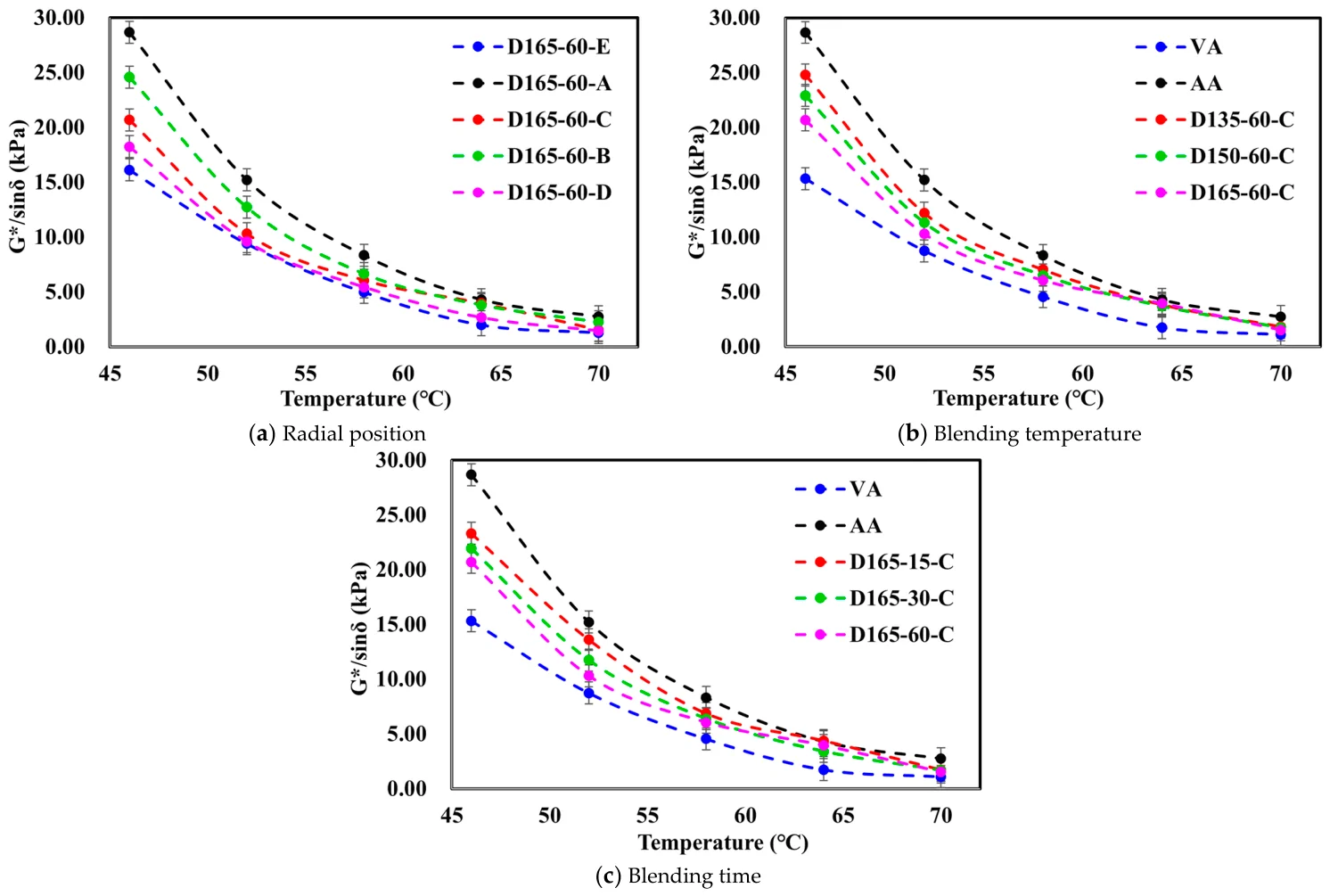
Temperature-sweep results under different conditions: (**a**) radial positions; (**b**) blending temperatures; (**c**) blending times.

Comparative analyses were conducted in terms of radial position, blending temper-ature, and blending time. Variations in the rutting factor within the interfacial region at different radial positions and under different temperature–time conditions were com-pared to evaluate the effect of interfacial diffusion between the virgin and aged asphalt binders on resistance to permanent deformation at high temperatures. The results are presented in Figure 7.

As shown in Figure 7a, the rutting factors at all radial positions of specimen D165-60 decreased significantly with increasing test temperature. At 46 °C, the rutting factor at Region A was 77.8% higher than that at Region E, indicating that pronounced heterogeneity remained within the specimen after blending at 165 °C for 60 min. This finding indicates that mutual diffusion between the virgin and aged asphalt binders occurred primarily in the region adjacent to the interface, whereas regions farther from the interface retained more of their original rheological characteristics. Figure 7a,b together show that increasing the blending temperature markedly reduced the rheological differences between the virgin and aged asphalt binders within the interfacial region. At 46 °C, the rutting factor of D165-60-C was 20.7 kPa, which was 27.8% lower than that of AA but remained 35.0% higher than that of VA, indicating that a transition region with characteristics of both binders had formed at Region C. When the blending temperature increased from 135 to 165 °C, the rutting factor at Region C decreased by 16.6%, indicating that increasing temperature enhanced component migration and interactions across the interface. Figure 7c further shows that extending the blending time promoted the homogenization of rheological properties within the interfacial region. At 165 °C, when the blending time was extended from 15 to 60 min, the rutting factor at Region C decreased from 23.31 to 20.68 kPa at 46 °C, corresponding to a reduction of 11.3%, and decreased by 24.3% at 52 °C. These results indicate that, with increasing blending temperature and isothermal treatment time, the rutting factor within the interfacial region generally decreased and gradually approached that of the virgin asphalt binder, demonstrating that increases in temperature and time facilitated material migration and the homogenization of rheological properties between the virgin and aged asphalt binders.

### 3.2. LAS Results

**Figure 8:**
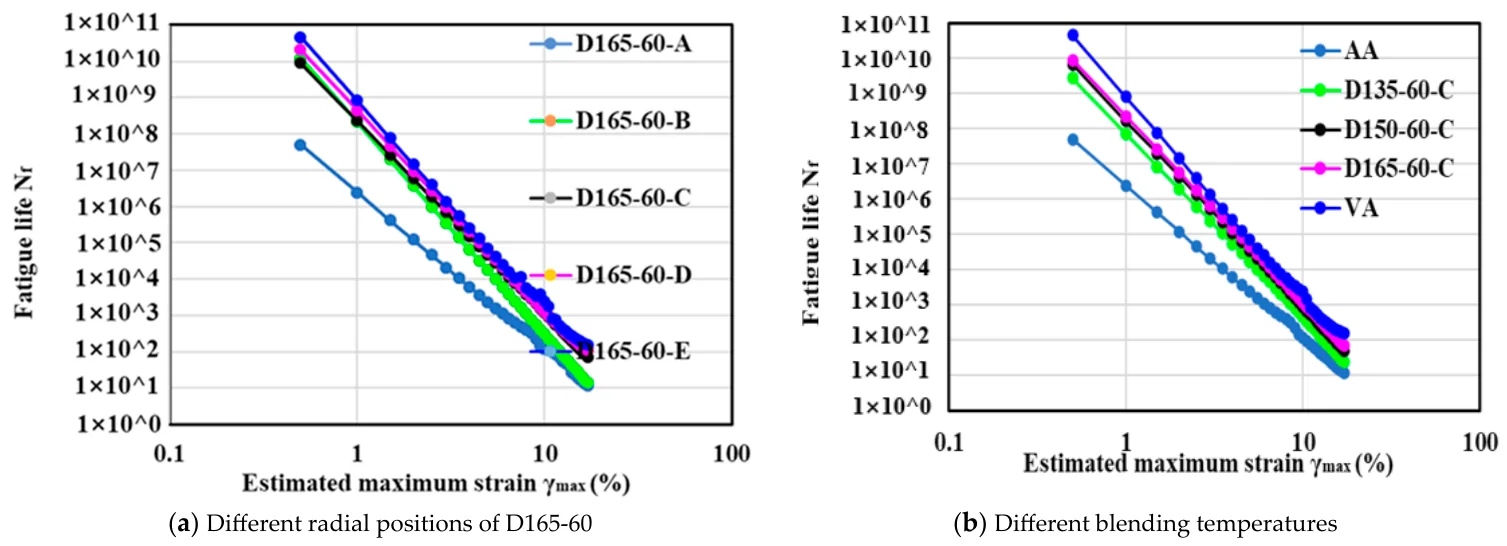

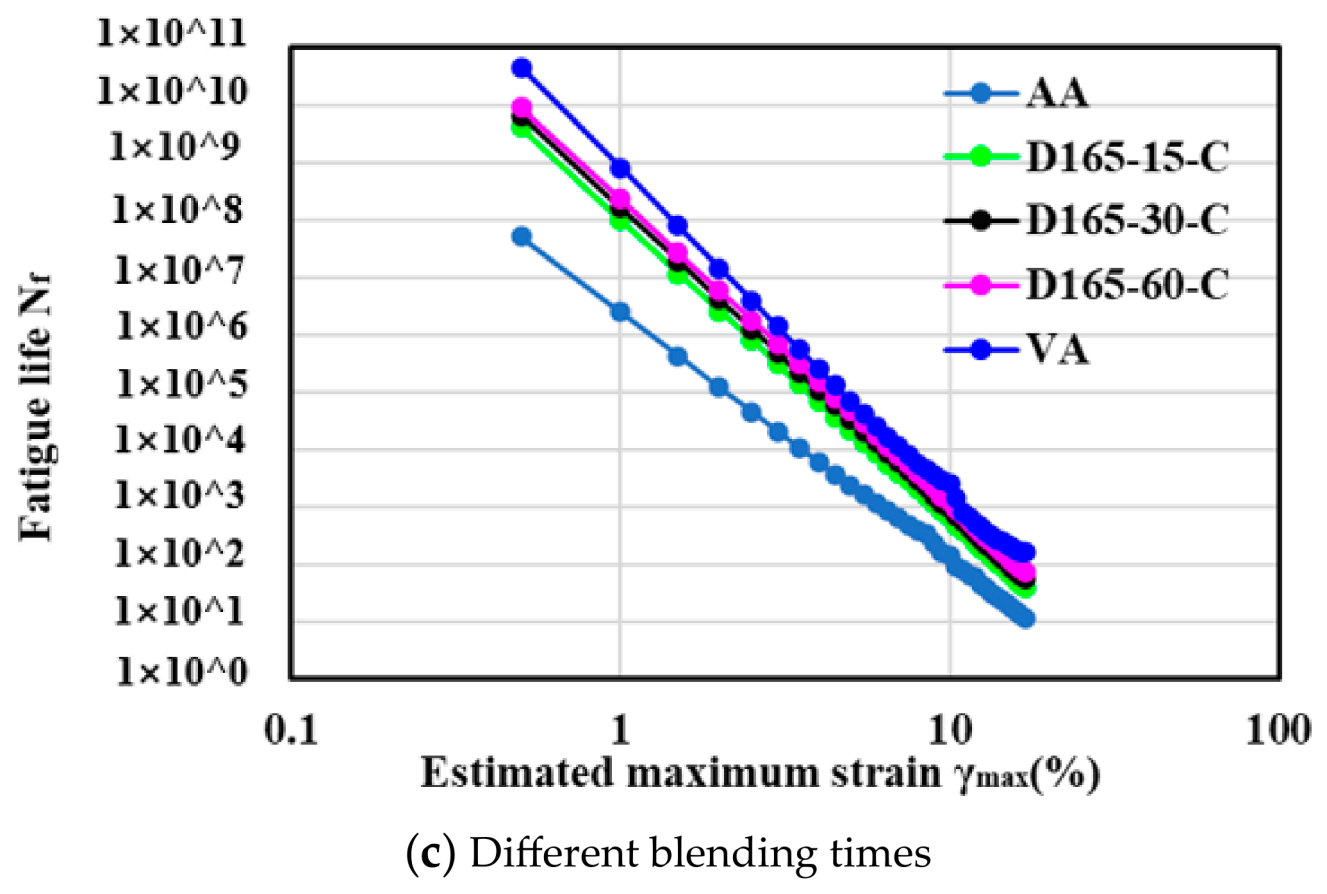
*N*_f_–*γ*_max_ curves under different conditions: (**a**) radial positions of D165-60; (**b**) blending temperatures; (**c**) blending times.

To evaluate the fatigue damage characteristics of the interfacial region after blending between the virgin and aged asphalt binders, LAS tests were used to determine fatigue life at different strain levels and establish the relationship between fatigue life and maximum shear strain. The fatigue responses at different radial positions and under different blending temperatures and times were compared to analyze the effect of diffusion between the virgin and aged asphalt binders on fatigue performance. The results are presented in Figure 8.

As shown in Figure 8a, fatigue life differed markedly among the radial positions under the D165-60 condition. As the sampling position shifted from the aged asphalt binder toward the virgin asphalt binder, fatigue life gradually increased in the following order: D165-60-A < D165-60-B < D165-60-C < D165-60-D < D165-60-E. This result indicates that the diffusion of virgin asphalt binder components into the interfacial region effectively improved the resistance of the aged-binder region to fatigue damage. Nevertheless, even after blending at 165 °C for 60 min, pronounced differences in fatigue performance remained among the radial positions, indicating that diffusion between the virgin and aged asphalt binders promoted performance recovery but did not result in complete homogenization. Figure 8b further illustrates the effect of blending temperature on interfacial fatigue performance. At the same blending time, increasing the blending temperature significantly increased fatigue life within the interfacial region. At a strain level of 5%, the fatigue lives of specimens D135-60-C, D150-60-C, and D165-60-C were 1.68 × 10^4^, 3.50 × 10^4^, and 4.60 × 10^4^, respectively, with the value at 165 °C being 173.8% higher than that at 135 °C. This increase was primarily attributable to the enhanced thermal motion of asphalt binder molecules at higher temperatures, which promoted the migration and rearrangement of virgin- and aged-binder components within the interfacial region and thereby reduced the adverse effect of the aged asphalt binder on fatigue performance.

Figure 8c shows the effect of blending time on fatigue performance. At the same blending temperature, fatigue life increased continuously as the blending time was extended from 15 to 60 min, although the magnitude of the increase gradually diminished. At a strain level of 5%, the fatigue lives of D165-15-C, D165-30-C, and D165-60-C were 2.34 × 10^4^, 3.30 × 10^4^, and 4.60 × 10^4^, respectively, with the value at 60 min being 96.6% higher than that at 15 min. These results indicate that extending the blending time promoted interfacial diffusion; however, as diffusion gradually became more complete, the additional performance improvement obtained by further extending the blending time diminished.

### 3.3. TLC-FID Results

**Figure 9:**
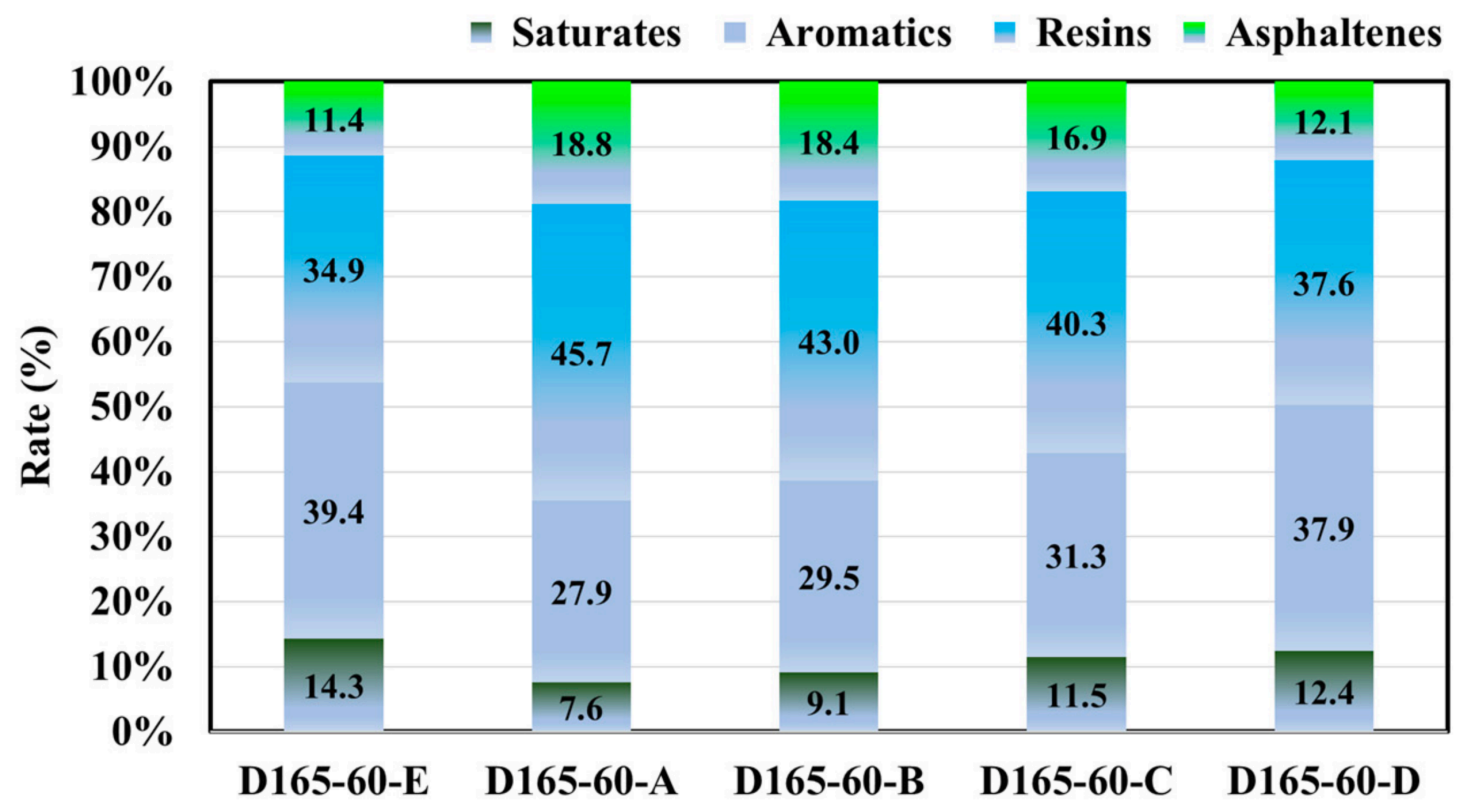
SARA fractions at different radial positions.

A four-component (SARA) analysis was conducted on samples obtained from different radial positions under the D165-60 condition, and the results are presented in Figure 9.

As shown in Figure 9, the SARA compositions of samples obtained from different radial positions differed markedly. The aged asphalt binder region exhibited higher contents of heavy components, with asphaltene and resin contents of 18.8% and 45.7%, respectively, whereas the saturate and aromatic contents were only 7.6% and 27.9%, respectively. In contrast, the saturate and aromatic contents in the virgin asphalt binder increased to 14.3% and 39.4%, respectively, while the resin and asphaltene contents decreased to 34.9% and 11.4%, respectively. These differences indicate that the enrichment of heavy components caused by aging was retained after blending, whereas the relatively active light components in the virgin asphalt binder migrated toward the aged-binder region. Further analysis of the radial variation in composition showed that, as the sampling position shifted from the central region of the aged asphalt binder toward the outward diffusion region, the aromatic content generally increased, whereas the asphaltene content gradually decreased. Specifically, the aromatic content increased from 27.9% to 39.4%, an increase of 41.2%. As aromatics serve as the dispersion medium for asphaltene aggregates, their increase can improve the dispersion of asphaltene micelles and thereby reduce the brittleness of the aged asphalt binder. This finding indicates that the thermal blending process involved not only simple physical contact but also structural adjustment of the asphalt binder components. The compositions of the interfacial transition regions D165-60-B, D165-60-C, and D165-60-D lay between those of the two end materials and exhibited continuous variation. This gradient distribution demonstrates the pronounced radial diffusion characteristics of blending between the virgin and aged asphalt binders. Rather than forming an abrupt interface, the interfacial region developed into a component transition layer of finite thickness.

### 3.4. Fluorescence Microscopy Results

**Figure 10:**
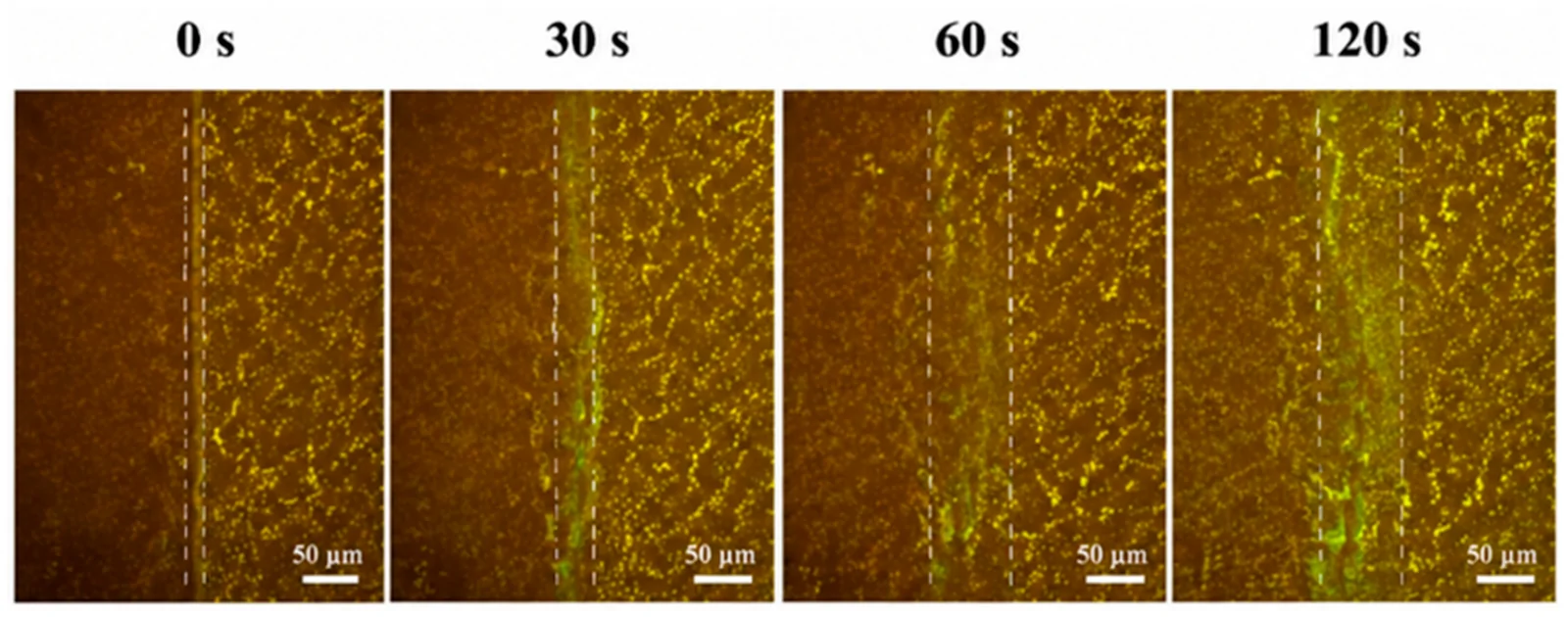
Fluorescence microscopy results for the virgin and aged asphalt binders.

Fluorescence microscopy was used to observe the interfacial morphology of thin layers of the virgin and aged asphalt binders after thermal treatment at 165 °C for 0, 30, 60, and 120 s, as shown in Figure 10. This test was used to characterize the early morphological evolution of the interface and the migration and blending process between the virgin and aged asphalt binders.

As shown in Figure 10, at the initial blending stage of 0 s, a relatively distinct boundary existed between the virgin and aged asphalt binders, and the bright and dark regions were clearly separated. This observation indicates that no substantial component exchange had yet occurred between the two binders and that the interface remained highly discontinuous. When the blending time increased to 30 s, a fluorescence transition region gradually appeared near the interface, indicating that active components in the virgin asphalt binder began to diffuse toward the aged-binder side and that an initial mixed region formed between the two binders. At a blending time of 60 s, the interfacial transition region expanded further and the initially distinct boundary gradually became blurred, indicating that the range of molecular migration continued to expand and that component exchange across the interface was further enhanced. After the blending time was extended to 120 s, the fluorescence distribution within the interfacial region became more continuous and the width of the diffusion layer increased markedly, indicating that a relatively stable transition structure had formed between the virgin and aged asphalt binders. However, differences in fluorescence remained on the two sides of the interface, indicating that the system had not reached a completely homogeneous state under the current blending conditions. The variations in the fluorescence images demonstrate that blending between the virgin and aged asphalt binders was distinctly time-dependent. Thermal exposure initially promoted component migration near the interface, after which the diffusion region gradually expanded toward both sides. These results are consistent with the compositional gradients observed in the SARA analysis and the radial performance differences measured in the DSR and LAS tests, further confirming that blending between virgin and aged asphalt binders is intrinsically a progressive process controlled by inter-facial diffusion.

### 3.5. Model Validation

#### Density

**Figure 11:**
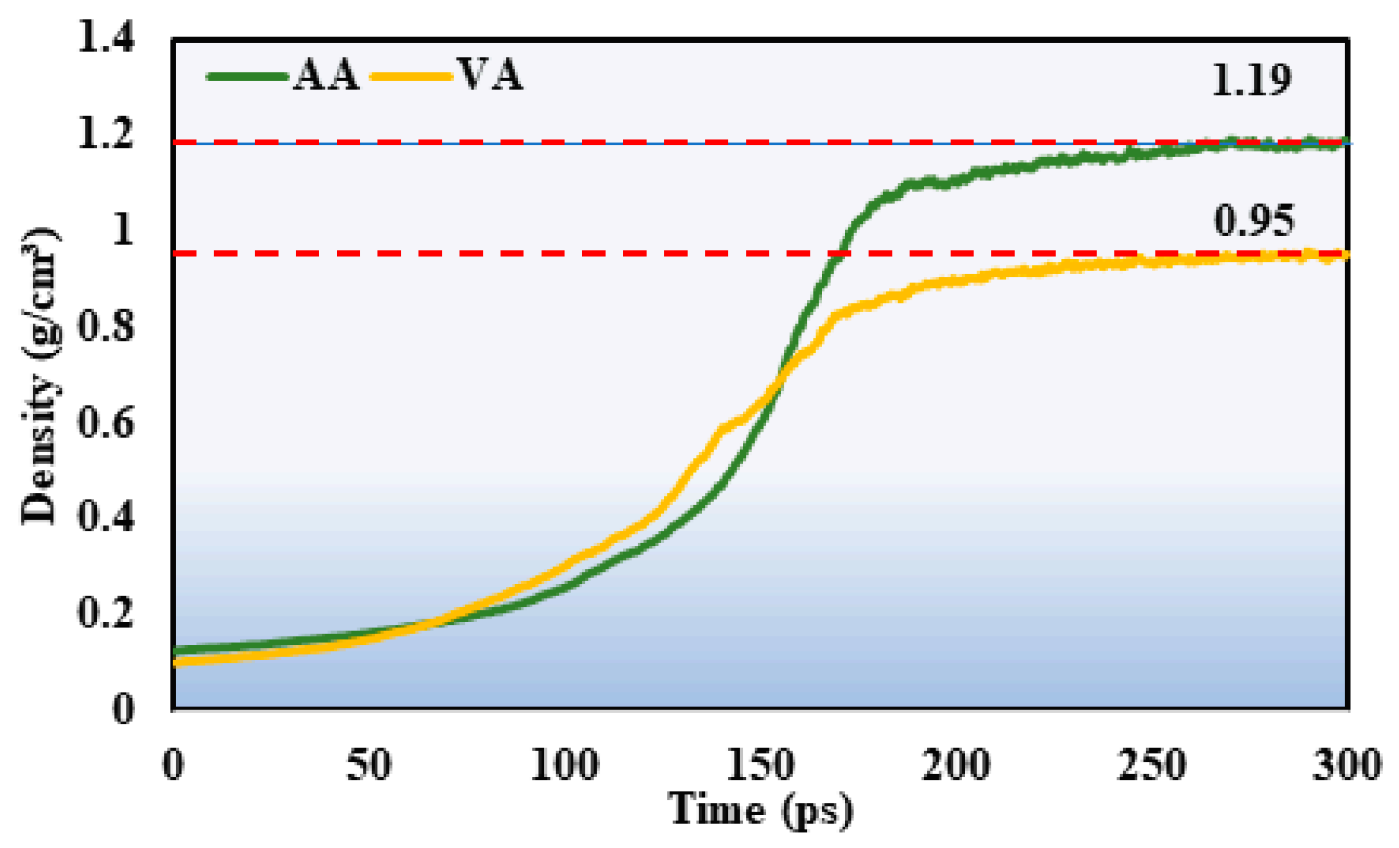
Densities of the two asphalt binder models.

As shown in Figure 11, the densities of both the VA and AA models gradually increased with simulation time and tended to stabilize after 200 ps, indicating that the systems had reached relatively stable states after NPT equilibration. After equilibration, the densities of the VA and AA models stabilized at approximately 0.95 and 1.19 g/cm^3^, respectively. In their molecular dynamics investigation of asphalt binders at different aging states, Qu et al. [26] likewise used the stable density after NPT equilibration as an important indicator for validating the molecular models. They also noted that aging changes the density and molecular packing state of an asphalt binder system, demonstrating that density convergence is commonly used to evaluate the reliability of asphalt binder molecular models. In the present study, the equilibrium density of AA was higher than that of VA, indicating that the asphalt binder system developed a more compact molecular packing state after aging. This trend is consistent with previous molecular simulation studies of asphalt binder aging.

**Figure 12:**
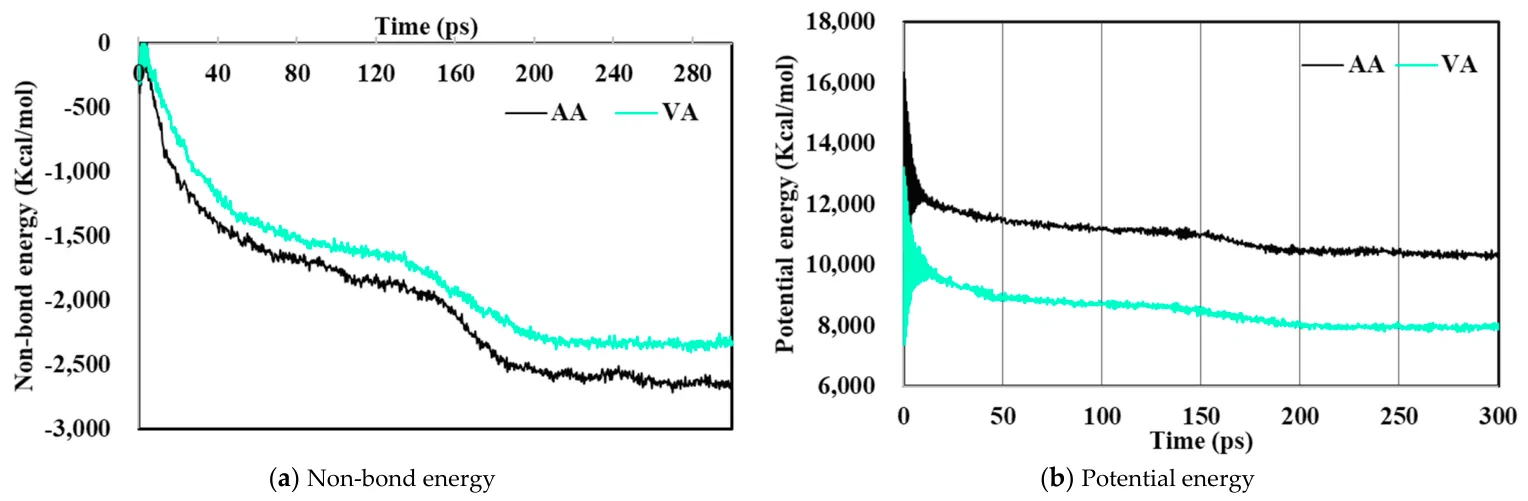

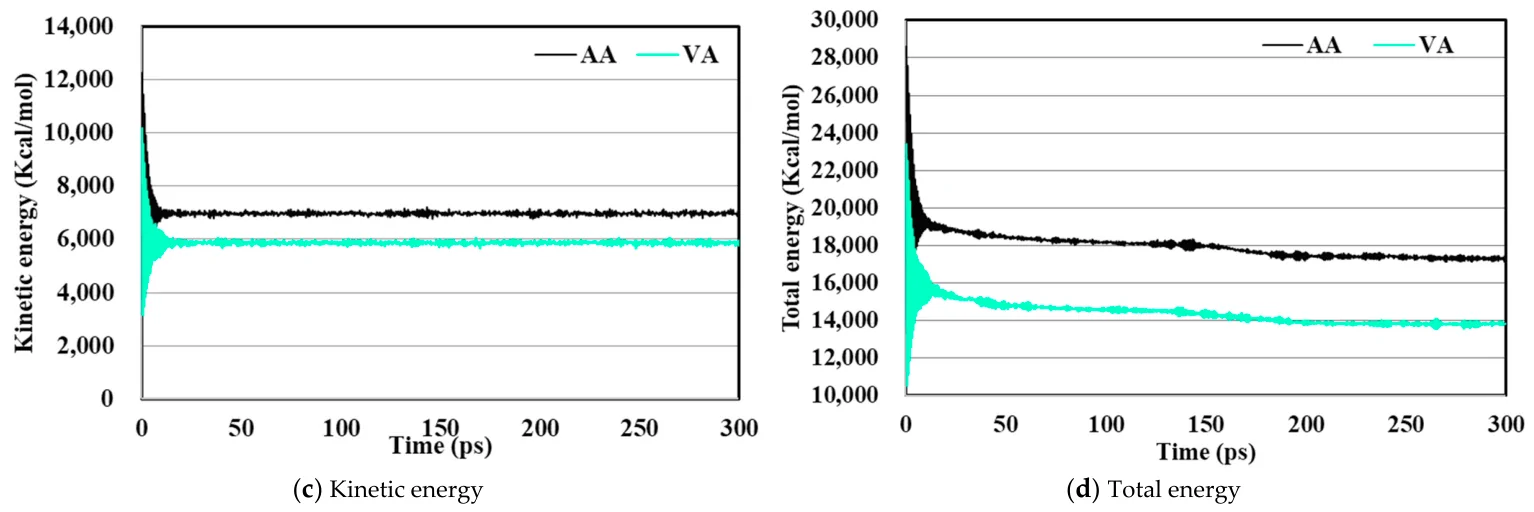
Energies of the asphalt binder models: (**a**) non-bond energy; (**b**) potential energy; (**c**) kinetic energy; (**d**) total energy.

To further validate the constructed molecular models of the virgin asphalt binder (VA) and aged asphalt binder (AA), the variations in nonbond energy, potential energy, kinetic energy, and total energy with simulation time during NPT equilibration were analyzed. As shown in Figure 12, the energy parameters of both models underwent substantial adjustment during the initial stage of simulation, after which the magnitude of their fluctuations gradually decreased. The nonbond energy continuously decreased as the system structure relaxed and became essentially stable after 200 ps. The potential and total energies exhibited similar convergence behavior and only fluctuated slightly around their stable values after 200 ps. Meanwhile, the kinetic energy rapidly reached a stable state within a relatively short period and exhibited only small fluctuations throughout the subsequent simulation, indicating stable temperature control. These results show that, after sufficient geometry optimization, annealing, and NPT dynamic equilibration, the VA and AA systems had moved beyond the high-energy states of their initial configurations and reached relatively stable thermodynamic equilibrium states.

### 3.6. Molecular Simulation Results for Virgin and Aged Asphalt Binders

#### 3.6.1. Mean Square Displacement

**Figure 13:**
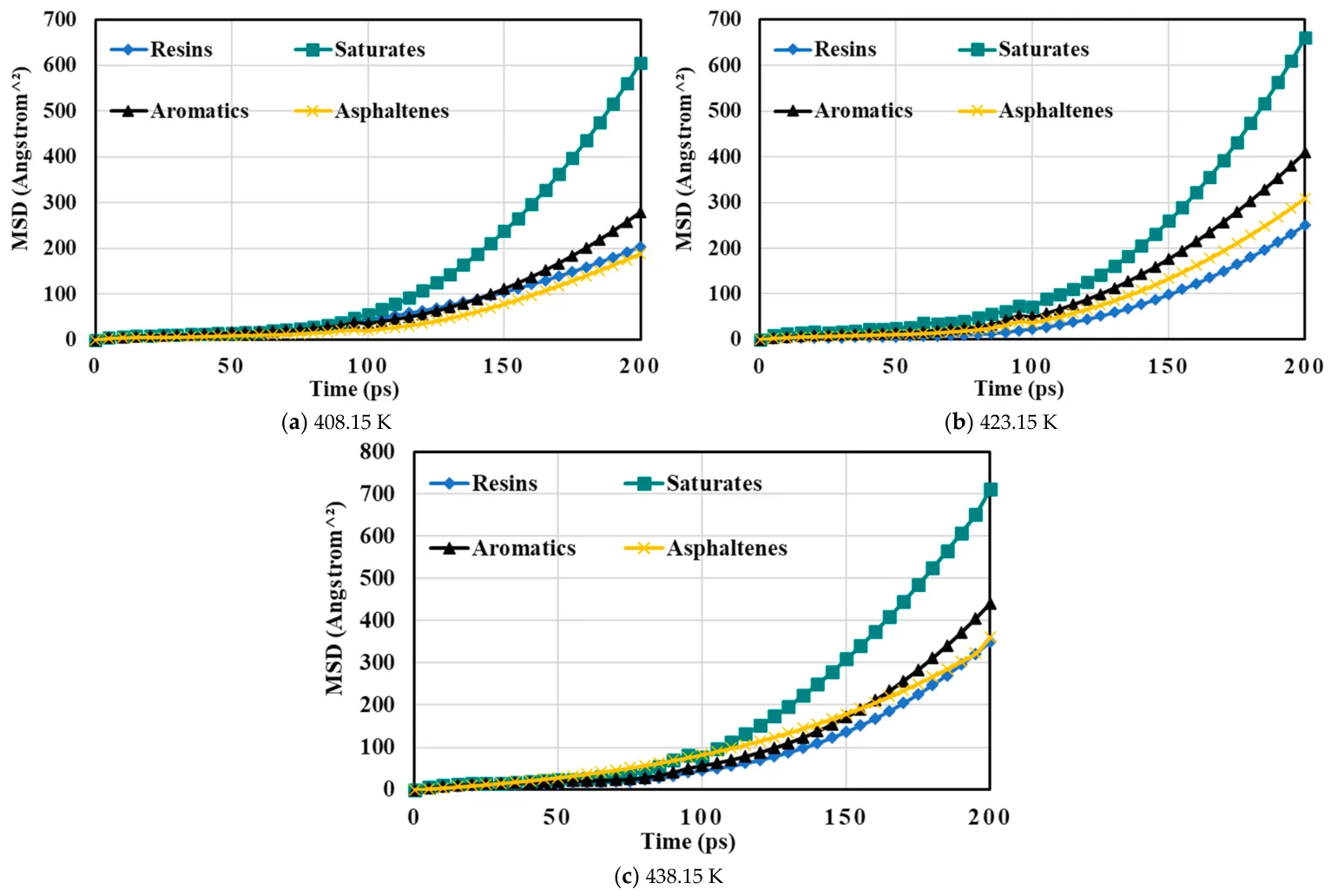
Mean square displacement of the asphalt binder components at different temperatures: (**a**) 408.15 K; (**b**) 423.15 K; (**c**) 438.15 K.

The mean square displacements of the saturates, aromatics, resins, and asphaltenes at 408.15, 423.15, and 438.15 K were calculated, and the results are presented in Figure 13. The MSD value reflects the extent of molecular displacement over the simulation time, with a higher value indicating greater molecular mobility of the corresponding component.

As shown in Figure 13, the MSD values of all components increased continuously with simulation time at 408.15, 423.15, and 438.15 K, indicating that the various components within the virgin asphalt binder possessed a certain degree of diffusivity. As the temperature increased, all MSD curves shifted markedly upward, demonstrating that increasing temperature enhanced molecular thermal motion and reduced the energy barriers to component migration. At 408.15 K and 200 ps, the MSD values of the saturates, aromatics, resins, and asphaltenes were 606.20, 278.25, 203.31, and 187.06 Å^2^, respectively. When the temperature increased to 438.15 K, the MSD values of these components increased markedly by approximately 17.4%, 58.2%, 71.7%, and 93.0%, respectively. The asphaltenes exhibited the largest increase in MSD, indicating that high temperatures effectively weakened the relatively strong intermolecular interactions among heavy components and enhanced their mobility.

#### 3.6.2. Relative Concentration

**Figure 14:**
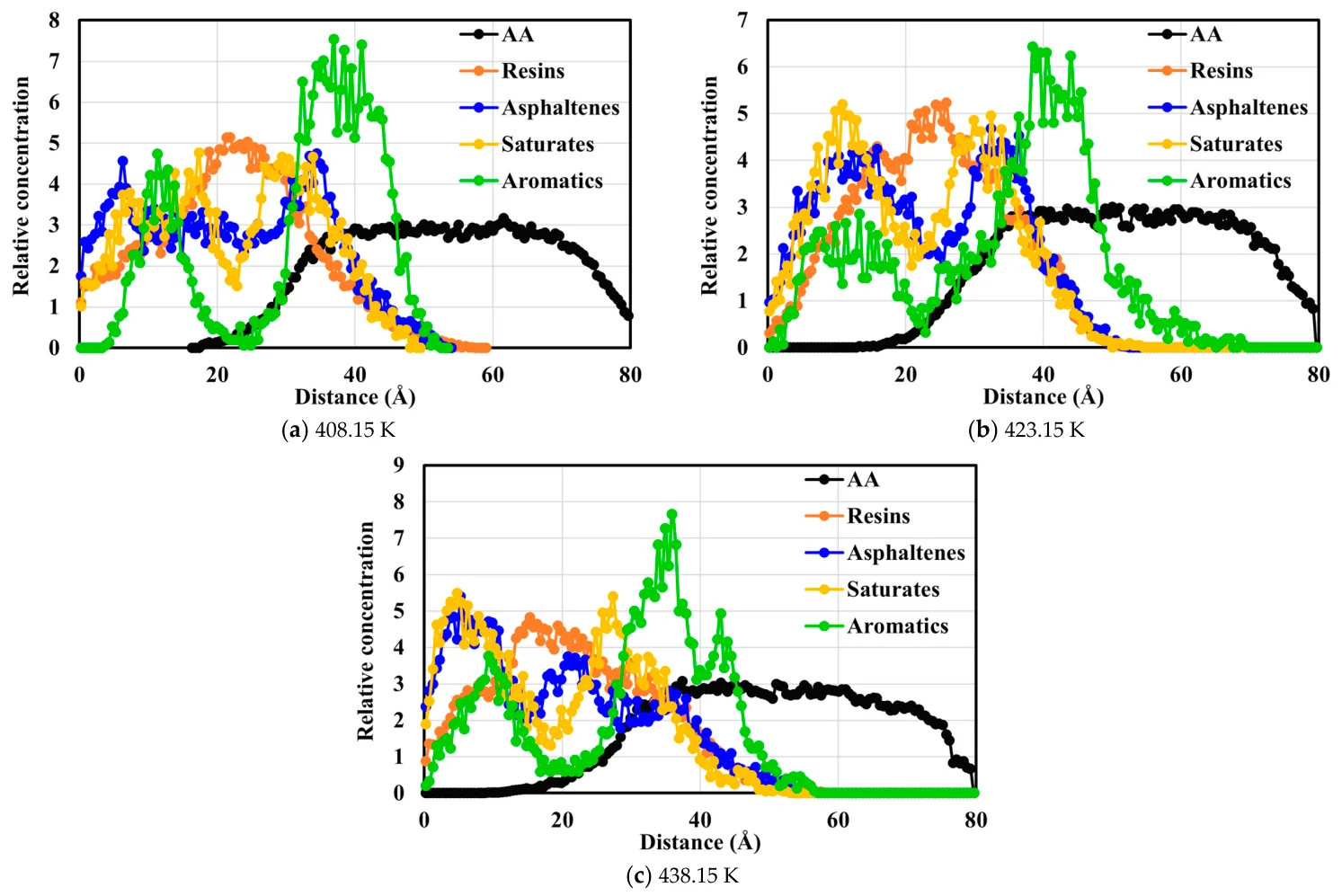
Relative concentrations of the asphalt binder components at different temperatures: (**a**) 408.15 K; (**b**) 423.15 K; (**c**) 438.15 K.

Figure 14 presents the relative concentration distributions of the components within the interfacial region between the virgin and aged asphalt binders at 408.15, 423.15, and 438.15 K. The spatial distribution ranges and the degree of overlap within the interfacial region provide more meaningful indications of molecular migration behavior.

As shown in Figure 14, the components of the virgin asphalt binder were mainly distributed on the left side of the model, whereas the aged asphalt binder was mainly distributed on the right side. At 408.15 K, the components of the virgin asphalt binder and the aged asphalt binder still exhibited relatively distinct spatial separation, with only a limited degree of concentration overlap near the interface. This indicates that interfacial blending was primarily at an initial stage of molecular interdiffusion at this temperature. As the temperature increased to 423.15 and 438.15 K, the relative concentration distributions of the aged asphalt binder molecules gradually extended toward the virgin-binder side, while the distribution ranges of the components within the virgin asphalt binder also changed markedly near the interface, indicating enhanced molecular interpenetration and component exchange across the interface. To quantitatively describe the diffusion of the asphalt binder components, the position at which the relative concentration of AA reached 0.5 was used as a reference. This position shifted from 24.88 Å at 408.15 K to 23.37 Å at 423.15 K and 21.86 Å at 438.15 K, corresponding to a cumulative displacement of 3.02 Å toward the virgin-binder side. This result indicates that, as the temperature increased, the migration range of the aged asphalt binder molecules into the virgin-binder region gradually expanded, accompanied by an increased degree of molecular overlap within the interfacial region. The different components exhibited distinct spatial migration behaviors. The aromatics showed a relatively pronounced distribution across the interface, whereas heavy components such as resins and asphaltenes predominantly underwent redistribution and gradual migration near the interface, reflecting the important effects of molecular structure and intermolecular interactions on diffusion behavior. Together with the preceding MSD analysis, these results demonstrate that increasing temperature enhanced the molecular mobility of all components and provided more favorable kinetic conditions for crossing the initial interface and participating in component exchange. Consequently, the virgin and aged asphalt binders gradually evolved from an initially distinctly layered state into an interfacial transition state with a broader range of spatial overlap. These molecular-scale changes demonstrate that blending between the virgin and aged asphalt binders was not a simple macroscopic mixing process, but rather a thermally driven process jointly governed by enhanced molecular thermal motion, diffusion across the interface, component redistribution, and local diffusion-induced restructuring.

## 4. Conclusions

This study focused on interfacial diffusion between virgin and aged asphalt binders and its effects on fatigue performance. A concentric-circle interfacial blending system was constructed, and the spatial variations in asphalt binder rheological and fatigue properties under different blending states were investigated by varying the blending temperature, blending time, and radial sampling position. By integrating LAS, DSR, SARA fraction analysis, fluorescence microscopy, and molecular dynamics simulations, the blending process between the virgin and aged asphalt binders was elucidated in terms of component migration, interfacial evolution, and molecular motion, and the relationship between interfacial diffusion and fatigue performance was examined. The main conclusions are as follows:

1. Pronounced radial heterogeneity remained after blending between the virgin and aged asphalt binders. After treatment at 165 °C for 60 min, the rutting factors on the aged-binder and virgin-binder sides still differed by 77.8% at 46 °C, and the interfacial region exhibited distinct transition characteristics. Increasing the blending temperature and extending the blending time reduced the rheological differences within the interfacial region.
2. A clear correspondence was observed between the extent of diffusion between the virgin and aged asphalt binders and fatigue performance. Fatigue life generally increased from the aged-binder side toward the virgin-binder side and increased with blending temperature and time. At 5% strain, increasing the blending temperature from 135 to 165 °C increased the interfacial fatigue life by 173.8%, while extending the blending time from 15 to 60 min increased it by 96.6%. These results indicate that enhanced interfacial diffusion improved the local resistance to fatigue damage.
3. The SARA fractions and fluorescence morphology indicated that blending between the virgin and aged asphalt binders was accompanied by continuous component migration and interfacial evolution. From the aged-binder side toward the virgin-binder side, the aromatic content increased, the asphaltene content decreased, and the interfacial composition varied continuously. With extended thermal exposure, the initially distinct boundary gradually became blurred and a transition region formed, further confirming the progressive diffusion characteristics of blending between the virgin and aged asphalt binders.
4. The molecular dynamics results showed that increasing temperature significantly enhanced the molecular mobility and interfacial interpenetration of all components. In particular, the MSD of the asphaltenes increased by 93.0% from 408.15 to 438.15 K, while the spatial overlap between the virgin and aged asphalt binders expanded simultaneously. The molecular-scale results were consistent with the macroscopic experimental trends, indicating that temperature promoted blending between the virgin and aged asphalt binders by enhancing molecular migration.

This study mainly analyzed the effects of temperature and time on interfacial diffusion behavior using a laboratory-constructed virgin–aged asphalt binder interfacial system. Future research will further consider the complex interfacial conditions in actual RAP materials and asphalt mixtures and investigate the combined effects of factors such as RAP content and mixing conditions on the blending behavior between virgin and aged asphalt binders. Meanwhile, the degree of interfacial diffusion will be quantitatively characterized, and the relationship between the interfacial blending state and the macroscopic performance of recycled asphalt mixtures will be further established to enhance the applicability of the research results to practical recycled pavement engineering.

## Figures and Tables

**Table 1:** Technical properties of the Grade 90 base asphalt binder.

Test Item	Test Result	Technical Requirement
Penetration (25 °C, 100 g, 5 s)/0.1 mm	85	80–100
Softening Point/°C	47.1	≥45
Ductility (5 cm/min, 15 °C)/cm	>144	≥100
Dynamic Viscosity (60 °C)/(Pa·s)	182.3	≥160
Density (15 °C)/(g/cm^3^)	1.007	Measured

**Table 2:** Number of molecules in each asphalt binder component.

Compound Type	Molecular Formula	Relative Molecular Mass	Number of Molecules
Asphaltene-phenol	C_42_H_54_O	575	3
Asphaltene-pyrrole	C_66_H_81_N	888	2
Asphaltene-thiophene	C_51_H_62_S	707	3
Quinolinohopane	C_40_H_59_N	554	4
Thioisorenieratane	C_40_H_60_S	573	4
Benzobisbenzothiophene	C_18_H_10_S_2_	290	15
Pyridinohopane	C_36_H_57_N	504	4
Trimethylbenzeneoxane	C_29_H_50_O	415	5
PHPN	C_35_H_44_	465	11
DOCHN	C_30_H_46_	407	13
Squalane	C_30_H_62_	423	4
Hopane	C_35_H_62_	483	4

## Data Availability

The original contributions presented in the study are included in the article. Further inquiries can be directed to the corresponding author.
